# Embedded Research: Possibilities for Learning Health Systems: Comment on "‘We’re Not Providing the Best Care if We are Not on the Cutting Edge of Research’: A Research Impact Evaluation at a Regional Australian Hospital and Health Service"

**DOI:** 10.34172/ijhpm.2023.7694

**Published:** 2023-08-07

**Authors:** Mandy M. Archibald

**Affiliations:** College of Nursing, Rady Faculty of Health Sciences, University of Manitoba, Winnipeg, MB, Canada.

**Keywords:** Embedded Research, Mentorship, Clinician-Researcher, Clinician-Scientist, Research Impact, Learning Health Systems

## Abstract

Brown et al show that research investments in an organization with a research and translation mandate can make important gains for research impact across domains, including quality of care and patient outcomes. Their multi-stage mixed methods evaluation provides insight into research capacity development in rural health systems in Australia and draws attention towards persistent geographic inequities. In extension of this important contribution, here, a focus on the "what and the why" of embedded research is offered. Specific attention is paid to the sustainability potentials of systematized data capture systems, funding-operational mandate alignments, researcher-scientist career pathways, and networked approaches to mentorship.

 In a timely article reflective of the need for renewed synergy between research practices and health services, Brown et al^1^ argue for embedded research to improve quality of care and patient outcomes. They present a 3 stage mixed methods evaluation study inclusive of realist logic to examine the impact of research investment from 2008-2018 at a regional hospital and health service in Queensland Australia, and identify contextual conditions influencing the attainment of impact markers. The evaluation seeks to gain insight into research capacity development by evaluating research investment, activity and impact at a regional hospital and health service in Australia.

 The authors highlight a need for research investment and capacity building in rural areas, citing that persistent geographic inequities exist. Consequently, these rural areas miss out on the benefits attributable to embedded research. While the authors identify a host of factors influencing its possible attainment with a focus on research capacity and investment, embedded research as a concept warrants additional attention. In complement to the work of Brown and colleagues, here, the question is presented: *What is embedded research?* And further, *why does it matter? *

## Embedded Research

 Embedded research is inherently pragmatic and involves the testing and subsequently the smooth integration of interventions into existing work flows, using standard care and ideally, routinely collected measures.^2^ Platt et al^3^ suggest embedded research involves a learning health system, emphasizing the use of embedded pragmatic trials that involve existing clinical staff instead of allocated research staff, use routinely collected data instead of research-specific metrics that require additional documentation, and are integrated into standard care. Such systems presume that (*a*) health systems routinely collect data on important impact markers pertinent to clinical care, practice, policy and patient outcomes; (*b*) health systems collect these data in a manner amendable to timely export and data standards, such as electronic health records (EHRs); (*c*) health professionals are in some way compensated for this work, because pragmatic trials still require research literacy and protocol compliance; (*d*) health professionals have the willingness to become researchers in this capacity, and that (*e*) they have the research literacy to work in this capacity. Notably, embedded research must — in the current climate of complexity and change mandating adaptation — account for a more pluralistic view of research and evidence.^4^ The current article by Brown et al^1^ speaks to some of these critical attributes. Perhaps most notable in their argument is the need for research capacity and for a systematic approach to research.

## Establishing a Systematic Approach to Research

 In their evaluation of the regional hospital and health service under investigation, Brown et al^1^ presented the key finding that an organization-wide, systematic process to enable research translation had not yet been established.This is likely to be the norm, rather than the exception in health systems worldwide. There are a number of factors influencing this, including the broader socio-cultural understanding of what research is, how it should be conducted, and what counts as quality evidence; questions of dominion, ownership and expertise around “specialty” skills such as research; growing awareness, stemming from the evidence based practice, knowledge translation, and implementation science movements that the mere presence of research does not itself result in practice and policy changes; and health system pressures, such as those presented by the novel coronavirus, which catalyzed the need for rapid assessment and implementation of effective treatments using a previously unparalleled global and embedded approach.^3-6^

 Critically, a systematic process for research translation requires the use of consistent and routinely collected measures within health systems. Indeed, while evaluation using frequently occurring and consistent measures is key to assessing the impact of research activities, routinely collected organizational metrics/measures of research capacity building are often absent from organizations. Processes to collect data on practice, policy and workforce and health impacts of research are also needed, and these need to be systematic. Brown et al^1^ rightly argue that this should be a priority moving forward.

 Using clinical data to improve patient care requires that the data points collected are meaningful, done so consistently, and are integrated as part of routine/standard care as much as is possible. Also critical is individuals’ capacity to operate well within organizations where such data is routinely collected. Ideally, process-oriented and values-based capacity building would accompany the integration of such data collection into EHRs wherever possible and include quality data standards to create systems ready for rapid-cycle feedback and improvement: ie, research ready, learning health systems.^2^ This is in part, the research infrastructure that Brown and colleagues identify as a critical component of their evaluation, yet goes beyond this to emphasize quality data metrics within each health system. Extending this view with the purview towards more multisite integration, enabling for instance, multi site trials, raises interest in the coordination of data capture within aligned technological systems (eg, EHRs), to avoid the need to reconfigure programming and data codes for each participating health system.^2^ As such, while Brown and colleagues^1^ emphasize the persistent geographic inequities that result from “missing out on the health and health system benefits attributable to embedded research” (p. 1), we are reminded that hospitals and health systems in major cities have in many cases not yet coordinated their data capture systems, impeding the learning potentials made possible through embedded research. Brown et al^1^ do well to draw attention to the under researched area of research capacity development in non-metropolitan settings in Australia.

## Research Capacity and Literacy as Critical to Research Capacity and Embedded Research Systems

 Research capacity is a broad term inclusive of such concepts as research infrastructure, research literacy, resources and other components that improve an organization’s ability to conduct research. The research capacity building process emphasizes developing sustainable abilities — in individuals and/or organizations — to conduct quality research.^6^ Indeed, research capacity is a multi-level concept, with relevant individual, organizational, and health system level applications. From an individual perspective, the adequate presence of on-the-ground personnel (eg, clinicians) with the time, support, interest/desire and know-how to conduct research is paramount, as is the “research literacy” of staff as discussed by Brown et al.^1^ Such components are integral to creating, and ultimately sustaining, embedded research systems.

 Through their evaluation, Brown et al^1^ identified a number of barriers to research literacy and capacity, such as managerial awareness of the time required to conduct research, the need for time compensation to off set clinical time with research time as well as protected time to gain research know-how, for example. Finding ways to increase – as Brown and colleagues^1^ refer to it – the “research literacy” of staff is indeed paramount to creating embedded and learning health systems. How to accomplish this objective, when clinical staff are already stretched within resource-constrained health systems? This creates opportunities for boundary spanning and capacity building roles, such as those of the knowledge broker and local champions, and also for dual expertise roles, such as those of the clinician-researcher.

 The clinician-researcher or clinician-scientist career pathway hold marked potential for improving institutional research capacity and the embeddedness of research.^7,8^ It is one avenue with targeted awareness and support at the trainee and early career levels in Canada, the United States and the Netherlands, for instance, as exemplified through funding and training opportunities provided by the Canadian Child Health Clinician Scientist and Training Upcoming Leaders in Pediatric Science programs, for example. The clinician-scientist career pathway is considered by some to be indispensable to the future of evidence-informed healthcare, particularly in light of collaborative and interdisciplinary necessities.^7^ While Brown et al^1^ identified that clear clinician-researcher career pathways are critical to attaining the goal of becoming a leading hospital research institute, threats to the physician-researcher pipeline have been reported, and the pathways for allied-health clinician scientists remain notably underdeveloped.^7,9^ Investments for clinician-scientists must acknowledge the prolongation and intensity of training required to gain both clinical and research proficiency, the personal work/life demands accompanying the training and career pathways, and the importance of mentorship for allied-health and physician-scientists, which are often at a shortfall due to a shortage of comparable faculty to prepare the next generation.^9^

 Leadership in health systems needs to think creatively and with a sense of urgency about these career pathways, as well as the impact of ­mentorship on research capacity. Mentorship, particularly in consideration of rural hospitals and health systems, should leverage the importance of networks that are gaining recognition within the complexity science literature.^5,10,11^ A mechanism to create links and enable mentorship networks across disciplinary and geographic boundaries would be advantageous, if not necessary, to advancing a sustainable approach to clinical-research mentorship. Empirical literature on networked approaches to mentorship, including the Developmental Network model of mentorship for instance, should inform organizational initiatives.^11^ Recognizing health professional educational training and employment as a continuum, a culture of research mentorship and opportunities for horizontal mentorship between peers should be established throughout training in the health sciences, to foster a collaborative research approach, provide social supports, candid advice, as well as early exposure to and aptitude for research.^10-13^

## Finances, Investment, and Mandate Alignment

 Financial investment in research made important impact gains in Brown and colleagues’ evaluation study.^1^ Yet, a major contextual barrier to research engagement that the authors identified was that research was not directly incentivised through the existing health system-funding model or service agreements. This points to a systemic issue that without creative resolution, will continue to thwart the true integration and embeddedness of research into health systems. As such, while Brown et al^1^ go on to speak about the alignment of funding and research priorities, it is also apparent how embedded research may compete with operational imperatives.^3^

 Platt et al^3^ offer 4 possible solutions to overcome barriers associated with costs, competing research and operational imperatives, and low research participation. These include that funders (1) reimburse health systems for costs of hosting trials; (2) establish research infrastructure in highly engaged systems; (3) shift the burden of research administration, often to coordinating centres; and (4) increase public awareness and reputational benefits associated with systematic research. Notably, the establishment of research infrastructure could also be targeted towards regional health authorities and health systems to help overcome the identified inequities. Increasing public awareness about the benefits of systematic research is aligned with what Brown et al^1^ identified as the external expectation to become involved in research, a pertinent example of a professional driver for research capacity and engagement.

 While Brown et al^1^ identified relevant broad contextual factors influencing research capacity, further attention to the dramatic shifts in the economics of healthcare worldwide is also warranted.^9^ In Canada, much of the economic debate — catalyzed by the COVID-19 pandemic — has focused upon policy reform in previously neglected areas, such as long term care and pharmacare.^14^ Rapid growth of health care expenditures is considered one of the most important economic trends in America, and it is within this context that research investment must be considered. Higgins^15^ and others have argued that investment in health research saves lives, and that current investments of the New Zealand government of 0.6%-0.8% of health spending into research is well below the estimated 2.4% required to improve health outcomes, including outcomes at the population health level. Positioning health research investment as a public health issue is necessary to garner public support; locating this attention towards research capacity investments as embedded in health systems is critical to maximizing the impact return of health research expenditures.

## Conclusion

 Research investments in an organization with a research and translation mandate can make important gains for research impact across domains, but the pathways are highly context dependent and contingent on a multitude of individual, organizational, and macro-level factors. Research capacity building aspirations should be directed towards creating embedded, learning health systems, reflective of research literacy and continued investments in resources spanning personnel, training, workflow, technological systems, and portfolio commitments, as examples. Fundamental however, is the need for a value and operational alignment; namely, that the support for research within institutions needs to match what institutions do to support it (eg, fund research activities, reduce service requirements). While these formidable challenges present opportunities for all health systems, attention towards the unique circumstances of regional authorities and health systems is particularly warranted.

## Ethical issues

 Not applicable.

## Competing interests

 Author declares that she has no competing interests.

## References

[1] Brown A, Edelman A, Pain T, Larkins S, Harvey G (2022). “We’re not providing the best care if we are not on the cutting edge of research”: a research impact evaluation at a regional Australian hospital and health service. Int J Health Policy Manag.

[2] Richesson RL (2020). Learning health systems, embedded research, and data standards-recommendations for healthcare system leaders. JAMIA Open.

[3] Platt R, Simon GE, Hernandez AF (2021). Is learning worth the trouble? - Improving health care system participation in embedded research. N Engl J Med.

[4] Greenhalgh T, Fisman D, Cane DJ, Oliver M, Macintyre CR (2022). Adapt or die: how the pandemic made the shift from EBM to EBM+ more urgent. BMJ Evid Based Med.

[5] Kitson A, Brook A, Harvey G (2018). Using complexity and network concepts to inform healthcare knowledge translation. Int J Health Policy Manag.

[6] Matus J, Walker A, Mickan S (2018). Research capacity building frameworks for allied health professionals - a systematic review. BMC Health Serv Res.

[7] Rosenblum ND, Kluijtmans M, Ten Cate O (2016). Professional identity formation and the clinician-scientist: a paradigm for a clinical career combining two distinct disciplines. Acad Med.

[8] Boaz A, Hanney S, Jones T, Soper B (2015). Does the engagement of clinicians and organisations in research improve healthcare performance: a three-stage review. BMJ Open.

[9] National Institutes of Health. Physician-Scientist Workforce Working Group Report. June 2014. https://acd.od.nih.gov/documents/reports/PSW_Report_ACD_06042014.pdf. Accessed September 15, 2022. 10.1111/cts.12209 PMC5439807 25123835

[10] DeCastro R, Sambuco D, Ubel PA, Stewart A, Jagsi R (2013). Mentor networks in academic medicine: moving beyond a dyadic conception of mentoring for junior faculty researchers. Acad Med.

[11] Halvorson MA, Finney JW, Bi X (2015). The changing faces of mentorship: application of a developmental network framework in a health services research career development program. Clin Transl Sci.

[12] Munce SE, Archibald MM (2017). “The future of mixed methods: a five year projection to 2020” an early career perspective. J Mix Methods Res.

[13] Zheng DX (2022). The need for horizontal mentorship networks to facilitate medical students’ engagement in research. Acad Med.

[14] Marchildon GP, Tuohy CH (2021). Expanding health care coverage in Canada: a dramatic shift in the debate. Health Econ Policy Law.

[15] Higgins C. Health research saves lives. Eur J Public Health 2020;30(Suppl 5):ckaa166.1262. 10.1093/eurpub/ckaa166.1262.

